# Surgical indicators for obstetrics and family planning in routine health information systems: a landscape analysis

**DOI:** 10.1093/heapol/czaf052

**Published:** 2025-08-08

**Authors:** Maxine Pepper, Oona M R Campbell, Karen Levin, Renae Stafford, Louise Tina Day, Vandana Tripathi, Fatima Abacassamo, Jumare Abdulazeez, Djibril Kébé, Jocelyne Kibungu, Sita Millimono, Manoj Pal, Feno Rakotoarimanana, Fatoumata Korika Tounkara, Josee Uwamariya, Sujata Bijou, Jennifer Snell, Farhad Khan

**Affiliations:** Department of Infectious Disease Epidemiology and International Health, London School of Hygiene & Tropical Medicine, Keppel Street, London WC1E 7HT, United Kingdom; Department of Infectious Disease Epidemiology and International Health, London School of Hygiene & Tropical Medicine, Keppel Street, London WC1E 7HT, United Kingdom; EngenderHealth, 505 9th Street NW, Suite 601, Washington, DC 20004, United States; EngenderHealth, 505 9th Street NW, Suite 601, Washington, DC 20004, United States; Department of Infectious Disease Epidemiology and International Health, London School of Hygiene & Tropical Medicine, Keppel Street, London WC1E 7HT, United Kingdom; EngenderHealth, 505 9th Street NW, Suite 601, Washington, DC 20004, United States; EngenderHealth, Rua José Mateus nr. 163, Maputo, Mozambique; EngenderHealth, Plot 247 Herbert Macaulay Way, Central Business District, Abuja, Nigeria; IntraHealth International, Sacré Cœur 1, Villa 8459, Résidence Amana, 7eme Étage, Dakar, Senegal; EngenderHealth, Concession Cotex, Bâtiment 5B – Avenue Colonel Mondjiba 63, Ngaliema, Kinshasa, Democratic Republic of Congo; EngenderHealth, 505 9th Street NW, Suite 601, Washington, DC 20004, United States; EngenderHealth, First Floor, Plot number 18, Ramnath House, Near Green Park Metro Station Gate No-2, Yusuf Sarai Community Centre, New Delhi 110049, India; Jhpiego, Immeuble Santa Lot II, 3rd Floor Antanimena, Antananarivo, Madagascar; IntraHealth International, BP 2243, Immeuble Tounkara, Hamdallaye ACI 2000, Rue 384, Porte 434, Bamako, Mali; IntraHealth International, Golden Plaza, 3rd floor, KG 546 Street 1, Kacyiru, Kigali, Rwanda; IntraHealth International, 6340 Quadrangle Drive, Suite 150, Chapel Hill, NC 27517, United States; IntraHealth International, 6340 Quadrangle Drive, Suite 150, Chapel Hill, NC 27517, United States; EngenderHealth, 505 9th Street NW, Suite 601, Washington, DC 20004, United States

**Keywords:** health information systems, indicators, surgery, caesarean, data, obstetrics, family planning, peripartum hysterectomy, health outcomes, surgical outcomes, procedures, female genital fistula care, long-acting reversible, reproductive sterilization

## Abstract

Strengthening use of high-quality data for surgical obstetrics and family planning is important for improving maternal and perinatal health outcomes. Routine health information systems (RHIS) represent an important data source for indicator tracking. This landscape analysis aims to describe and compare surgical obstetric and family planning indicators put forth by global multi-stakeholder groups and those that are currently captured in RHIS in nine low- and middle-income countries. The analysis focused on five indicator topics: (i) caesarean delivery, (ii) peripartum hysterectomy, (iii) female genital fistula care, (iv) insertion/removal of long-acting reversible contraception and male/female sterilization, and (v) the general surgical context. We examined 12 indicator lists developed by multi-stakeholder groups and RHIS documentation from the Democratic Republic of the Congo, Guinea, India, Madagascar, Mali, Mozambique, Nigeria, Rwanda, and Senegal. 29 multi-stakeholder and 104 country indicators (119 unique indicators) met our inclusion criteria, typically capturing service provision or service readiness. Indicators on post-surgical outcomes or complications were rarer. The reviewed multi-stakeholder lists did not include indicators on peripartum hysterectomy. At the country level, not all RHIS included fistula care or peripartum hysterectomy indicators and there were marked differences with regard to what indicators were included and the relative distribution of indicators across the indicator topics. Only 14 (48%) of the multi-stakeholder indicators were included in countries’ RHIS, with just two being tracked by all nine countries (caesarean deliveries and family planning users by modern method of contraception). There was a lack of standardized indicators for surgical obstetrics and family planning, and we noted typical RHIS challenges such as indicator profusion, duplication, vague indicator definitions, and measurement of composite or difficult-to-quantify concepts. Our findings suggest that there are opportunities to standardize and streamline prioritized measurement of surgical obstetric and family planning data for tracking with the ultimate aim of improving health services.

Key messages

- Routine health information system (RHIS) indicators can inform programme planning, but we know little about what is currently tracked for surgical obstetrics and family planning. We explored this routine indicator landscape for nine countries and across 12 global multi-stakeholder indicator lists.
- Our findings highlighted key gaps in routine indicator measurement for surgical obstetrics and family planning. We found a lack of global consensus on what indicators to measure, a discordance between indicators put forth by global multi-stakeholder groups and indicators included in country RHIS, and few indicators on peripartum hysterectomy or post-surgical outcomes and complications.
- Standardized RHIS indicators enable comparison over time and geographies. Reviewing and refining indicators for surgical obstetrics and family planning could ensure that actionable data are being collected. Such efforts could include prioritization exercises at the global and country levels, metadata definitions, and removal of redundant indicators to maximize data quality for use with minimal burden for health and data professionals.

## Introduction

Despite improvements in maternal and perinatal health, and increased use of family planning services (Haakenstad *et al.* 2022, World Health Organization *et al.* 2023), recent data show that maternal mortality declines have stalled (World Health Organization *et al.* 2023). Multi-faceted efforts are needed for countries to collectively meet the Sustainable Development Goals of reducing the global maternal mortality ratio to less than 70 per 100 000 live births and the global newborn mortality rate to less than 12 per 1000 live births by 2030. This requires scaling up core interventions such as antenatal care, institutional delivery, and skilled birth attendance (UNICEF Data 2022a, 2022b, World Health Organization *et al.* 2023), and a shift in the focus of maternal and newborn health programming towards improving quality of care (Kruk *et al.* 2018) and enhancing hospital-based services (Boerma *et al.* 2023).

Strengthening surgical interventions in obstetrics and family planning is crucial for driving these efforts forward. Surgical care is increasingly recognized as an integral health system component, essential for addressing a wide range of health challenges (Meara *et al.* 2015). In obstetrics, caesarean delivery is a key surgical intervention and has been a signal function for comprehensive emergency obstetric and newborn care since 1997 (Moxon *et al.* 2024). Caesarean section rates, particularly when stratified by wealth indices or Robson criteria, can be used to monitor both over- and underuse of this life-saving procedure. Other important surgical interventions in obstetrics and family planning include peripartum hysterectomy, fistula repair, insertion/removal of long-acting reversible contraception (LARCs) and male/female sterilization (permanent methods of contraception, PMs). However, access to surgical services is highly inequitable, with 97% of the population in low-income countries lacking timely access to safe, affordable surgical care (Alkire *et al.* 2015). Improved measurement is crucial for catalyzing health policy change, ensuring effective programme management, promoting timely quality improvement efforts, and enabling better programme evaluation, thereby leading to better health outcomes (Meara *et al.* 2015, World Health Organization 2015).

Routine health information systems (RHIS) are an important source for data on surgical obstetrics and family planning procedures. RHIS house regularly collected aggregate service delivery data from health facilities, sourced from health facility registers (MEASURE Evaluation, 2016). RHIS data are usually recorded by health workers and abstracted from registers onto summary forms. These data are transferred to district health offices and then aggregated and reported to the subnational and national levels (Amouzou *et al.* 2021). RHIS data are used at all levels of the health system to monitor service provision, guide policymaking, and plan programmes (World Health Organization 2023).

MOMENTUM Safe Surgery in Family Planning and Obstetrics (2020-2025) is a global project funded by the United States Agency for International Development to promote awareness of, equitable access to, and high quality of care for voluntary and consented safe surgeries within maternal health and family planning programmes. Recognizing the potential value of these data for programme planning and quality improvement, we carried out this study to describe and compare which surgical obstetric and family planning indicators were put forth by multi-stakeholder actors at the global level and which indicators were captured in the RHIS of nine low- and lower-middle income project implementation countries. The study focused on four categories of procedures: caesarean delivery, LARCs and PMs, female genital fistula care, and peripartum hysterectomy. Rather than identifying a set of ‘priority’ indicators, the purpose of the analysis is to provide an overview of listed indicators at country and global level, a crucial first step towards understanding the RHIS function. To our knowledge, this information has not yet been compiled, despite its relevance and potential for improving maternal health by tracking actionable indicators on surgical obstetrics and family planning.

## Materials and methods

This landscape analysis was designed to capture indicators on the four focus procedures as well as the wider surgical context (Table 1). Surgical context was incorporated as a fifth topic to capture cross-cutting data relevant to all four focus procedures and beyond.

**Table 1.** Classification of identified indicators.

Indicator topic	Indicator eligibility
**Indicators related to the four focus procedures**	
1. Caesarean delivery	Indicators were included if they: described the number of procedures carried out, quantified specific aspects of the procedure, and described outcomes related to procedure; described service readiness specific to the procedure of interest (e.g. number of providers trained in fistula repair, stock of specific long-acting contraceptives). Indicators were excluded if they: described the prevalence/aetiology of the health conditions leading to these procedures (e.g. cause of fistula).
2. Peripartum hysterectomy	
3. Fistula care	
4. LARCs and PMs (and other family planning indicators which included LARCs/PMs)	
**Indicators related to the wider surgical context**	
5. Surgical context	Indicators were included if they: described the broader context primarily relevant to surgery, including readiness for surgery (e.g. surgical workforce, operating theatre, anaesthetics), total number of any surgical procedure, and postsurgical outcomes; described umbrella concepts (e.g. ability to provide EmONC services) which provide relevant information about the context within which the focus procedures take place. Indicators were excluded if they: described individual surgical procedures (beyond the four focus procedures); described the broader facility context, not only specific to performing surgery (e.g. electricity, water and sanitation, infection prevention).

EmONC, Emergency Obstetric and Newborn Care; LARCs, long-acting reversible contraception; PMs, permanent methods of contraception.

**Figure 1.**
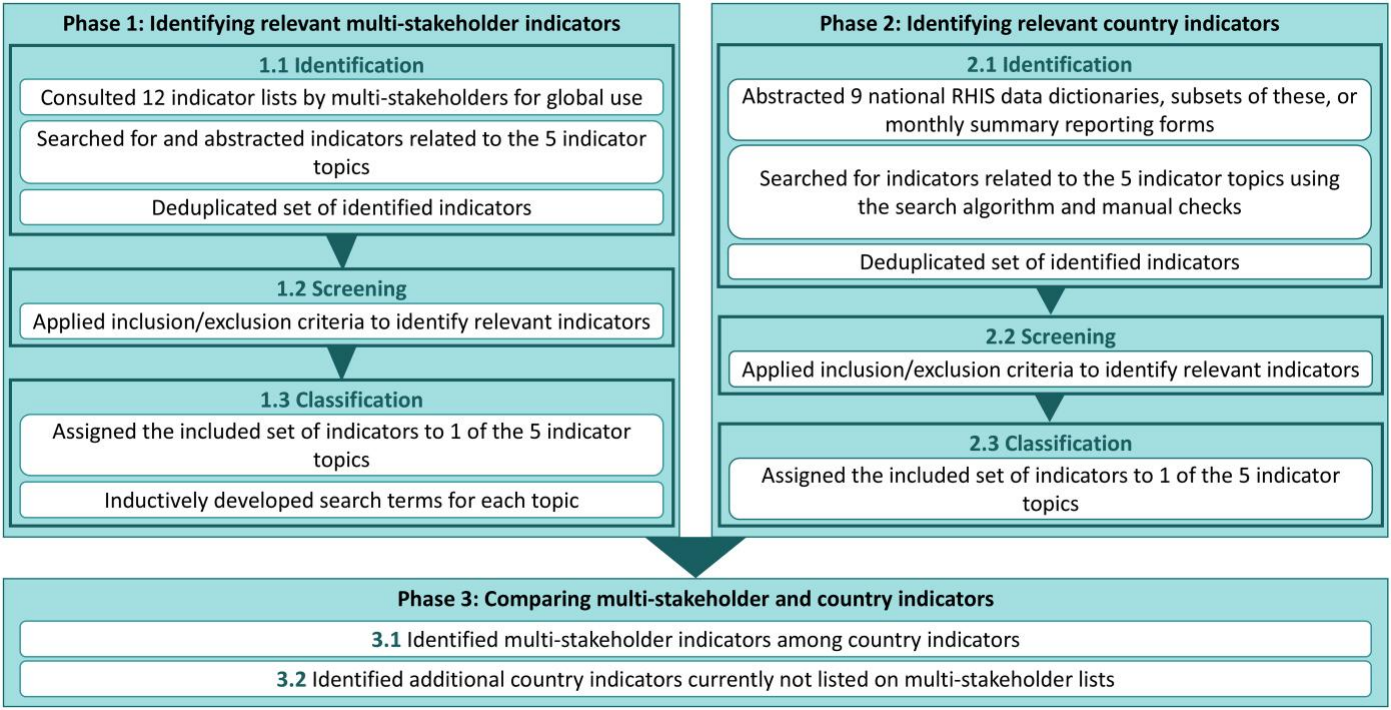
Overview of methods: the three phases comprising the landscape analysis. RHIS, routine health information systems.

We reviewed the indicators in three phases (Fig. 1). In Phase 1, we identified sets of indicators which were listed by multi-stakeholder groups. In Phase 2, we reviewed RHIS country indicator sources in nine countries: the Democratic Republic of the Congo (DRC), Guinea, India, Madagascar, Mali, Mozambique, Nigeria, Rwanda, and Senegal. And in Phase 3, we compared the multi-stakeholder and country indicators identified.

### Phase 1: identifying relevant multi-stakeholder indicators

#### Identification and extraction

From July to September 2022, we searched for indicator lists for global use which included indicators related to caesarean delivery, peripartum hysterectomy, fistula care, or family planning. We began with indicator lists developed by United Nations organizations. This yielded eight source documents (Every Woman Every Child 2016, Moran *et al.* 2016, World Health Organization 2016, 2018, 2020, 2021, Jolivet *et al.* 2018, World Health Organization and UNICEF 2019). Then, we searched for indicator lists developed as part of special initiatives related to the five indicator topics. This yielded another four source documents (FP2020, MEASURE Evaluation; Ngongo *et al.* 2015, Davies *et al.* 2021). The 12 source documents identified were reviewed by one author who abstracted indicators using Microsoft Excel. We adopted an approach aimed at capturing all indicators broadly related to our five indicator topics; in other words, an approach with high sensitivity (Table 1). Two authors reviewed the list of extracted indicators and deleted duplicative indicators which were similar in meaning.

#### Screening

We complemented our sensitive approach to indicator identification with a screening stage focused on increasing specificity. Indicators from multi-stakeholder lists were included if they (i) met the eligibility criteria outlined in Table 1 and (ii) were deemed measurable through RHIS (for detailed inclusion/exclusion criteria, see Supplementary material). Indicators were double-screened and additional team members reviewed screening decisions when needed.

#### Classification

The multi-stakeholder indicators we included were each assigned to one of the five indicator topics. For each topic, we developed a set of corresponding search terms (in English, French, and Portuguese; see Supplementary material) to aid the identification of similar country indicators in Phase 2.

### Phase 2: identifying relevant country indicators

#### Identification and extraction

In November and December of 2022, we sourced full RHIS data dictionaries for each of the nine countries. Where these were not available, we sought data dictionary subsets or lists capturing indicators which fell within the five indicator topics. If subsets of data dictionaries or indicator lists were not available, we sourced monthly summary reporting forms which health facilities submit to the district level for input into the RHIS. The monthly summary forms included data elements (i.e. a singular data dimension used as the numerator or denominator in the indicator) as opposed to indicators (i.e. mathematical operations of data elements, often a percentage or sum) (DHIS2 2021). Within the following sections, the term ‘indicator’ is used to refer to either data elements or indicators. We used Microsoft Excel to abstract the received country files and to create searchable indicator lists.

The prepared Excel lists were then searched for indicators which fell into the five indicator topics. To ensure high sensitivity, indicators were identified using a combination of a search strategy and manual checks. First, we ran a search based on the terms identified in Phase 1. Then, one author visually screened the lists to identify any other potentially relevant indicators which were missed by the algorithm. Before moving to the screening stage, one author reviewed the set of indicators identified by the algorithm and suggested indicators for deletion if they were clearly out of scope (e.g. blood tests for malaria, which would have been identified by a search term for ‘blood’ intended to pick up blood transfusions). No indicator was deleted unless reviewed by at least two authors. Lastly, two authors worked together to detect and delete duplicates among the identified country indicators.

#### Screening

As with the screening process in Phase 1, identified indicators were then screened against specified inclusion/exclusion criteria.

#### Classification

The final set of included country RHIS indicators was classified into the five indicator topics.

### Phase 3: comparing multi-stakeholder and country indicators

#### Identifying multi-stakeholder indicators among country indicators

We examined the eligible country indicators to ascertain whether they contained any of the included multi-stakeholder indicators. Two authors worked together to assess whether the country indicators were similar in meaning to any of the multi-stakeholder indicators. If a country recorded an indicator similar in meaning to a multi-stakeholder indicator but with less detail (e.g. lacked disaggregation), we still recorded it as a multi-stakeholder indicator collected in this country. Similarly, for multi-stakeholder indicators requiring calculation (e.g. percentages), we judged that a country was collecting this indicator if we found the required numerator in the country’s data dictionary. In our study, we often only had access to data element lists and many denominators (e.g. total live births) could be easily derived by aggregating relevant data points, such as summing caesarean and vaginal deliveries to obtain total deliveries. Since the absence of individual data elements did not exclude the possibility that an indicator’s denominator could be accurately computed, we focused on the primary data elements (i.e. numerators). In some instances, multiple indicators within a country mapped onto one multi-stakeholder indicator (e.g. multi-stakeholder indicator on family planning users stratified by method of contraception versus country RHIS including indicators for each method).

#### Identifying additional country indicators not listed on the multi-stakeholder indicator lists

Next, we sought to identify any other indicators related to the five indicator topics that were available across the nine countries’ RHIS. For each additional country indicator identified, two authors reviewed it, and, if applicable, matched it to indicators with similar meanings in other countries.

Combining the list of identified multi-stakeholder and additional country indicators, we created a full list of all unique indicators identified across the five indicator topics and their availability within the nine country RHIS.

## Results

Across the 12 indicator lists developed by multi-stakeholder groups, we identified 73 unique indicators broadly related to the five indicator topics of interest. 29 of these indicators met our eligibility criteria and were included in the analysis.

For three of the nine countries, we were able to source indicator lists: Guinea, Mali, and Senegal (Table 2). For the other six, we sourced data elements directly from RHIS health facility monthly summary forms (Madagascar, Nigeria, Rwanda, and Mozambique) or from existing data element lists (India, Nigeria). Across these documents, we identified 740 indicators broadly related to the five indicator topics of interest. 104 indicators met our eligibility criteria and were included in the analysis (see Supplementary Figure S1 for more details on the indicator identification and screening process).

**Table 2.** RHIS country indicator sources.

Country	Source type	Source name and year
DRC	Summary form	Canevas Mensuel de L’Hospital, 2016.
Guinea	Indicator list	Guide des Indicateurs du Secteur Santé, Développement Social et Promotion de la Famille, 2017.
India	Data element list	Data Item Wise HMIS Indicator, no date specified.
Madagascar	Summary form	Rapport Mensuel d’Activites des Centre Hospitalier de Référence de District Avec et Sans Chirurgie, 2022
Mali	Indicator list	Guide des Indicateurs du Secteur Santé, Développement Social et Promotion de la Famille, 2017 (supplemented with manual search for indicators in DHIS2)
Mozambique	Summary form	Saúde Materno-Infantil (SMI)-Resumo Mensal da Maternidade da Unidade Sanitária (US), 2022 and Resumo Mensal da US—SMI—Consulta De Saúde Reprodutiva/Planeamento Familiar, 2022
Nigeria	Data element list	National Health Management Information System, Health Facility Monthly Summary Form, 2019
Rwanda	Summary form	District Hospital Monthly HMIS Report, 2021.
Senegal	Indicator list	Matrice Globale des Indicateurs de la Santé de la Mère et de l'Enfant du Ministère de la Santé et de l'Action Sociale, 2022.

DRC, Democratic Republic of the Congo; HMIS, Health management information system.

### Multi-stakeholder indicators

The 29 eligible indicators put forth by multi-stakeholder groups covered four of the five indicator topics (caesarean: *n* = 6, fistula: *n* = 10, LARCs and PMs: *n* = 8, surgical context: *n* = 5). We did not identify any peripartum hysterectomy indicators.

For caesarean delivery, the multi-stakeholder indicators included indicators related to service provision (e.g. percent of deliveries by caesarean section), service readiness (e.g. adequate number of staff skilled in performing caesarean section, 24 h a day), and post-caesarean complications (e.g. severe systemic infection or sepsis after caesarean section).

For fistula care, the multi-stakeholder indicators included indicators related to service provision (e.g. number of fistula repairs by type), service readiness (e.g. doctors trained in obstetric fistula repairs), and outcomes of fistula repairs (e.g. number of discharged fistula repair patients not closed or with remaining incontinence).

For LARCs and PMs, the multi-stakeholder indicators included indicators related to service provision (e.g. family planning users by modern method of contraception) and service readiness (e.g. number of health providers trained in long acting and permanent services). We identified one indicator on contraceptive implant removal, but none on outcomes/complications related to insertion/removal of LARCs or female/male sterilization.

For the surgical context, the multi-stakeholder indicators included indicators related to surgical volume (e.g. total number of surgical procedures), facility designation (e.g. facilities offering comprehensive emergency obstetric and neonatal care), and surgical outcomes (e.g. deaths among patients undergoing surgical procedures). For a full list of the 29 multi-stakeholder indicators, see Supplementary Table S1.

Some of the multi-stakeholder indicator lists included similar indicators. However, despite being similar in meaning, the phrasing of the indicator definition differed across lists. For example, there were two different multi-stakeholder indicators on perioperative mortality (which we included as one indicator in the analysis): ‘All-cause death rate prior to discharge among patients having one or more procedures in an operating theatre during the relevant admission’ (World Health Organization 2018) versus ‘Deaths from all causes, before discharge (up to 30 days), in all patients who have received any anaesthesia for a procedure done in an operating theatre’ (Davies *et al.* 2021). Other multi-stakeholder indicators addressed important dimensions of care, but we judged that they would be very difficult to operationalize in practice as part of routine measurement. For example, an indicator such as ‘The health facility has an adequate number of staff skilled in performing caesarean section, 24 h a day’ (World Health Organization 2016) requires a definition of ‘adequate number’ in relation to workload, a measure of ‘skill’ and its maintenance, and a way of assessing ‘24 h a day’ in an ongoing fashion.

### Country routine health information system indicators

**Figure 2.**
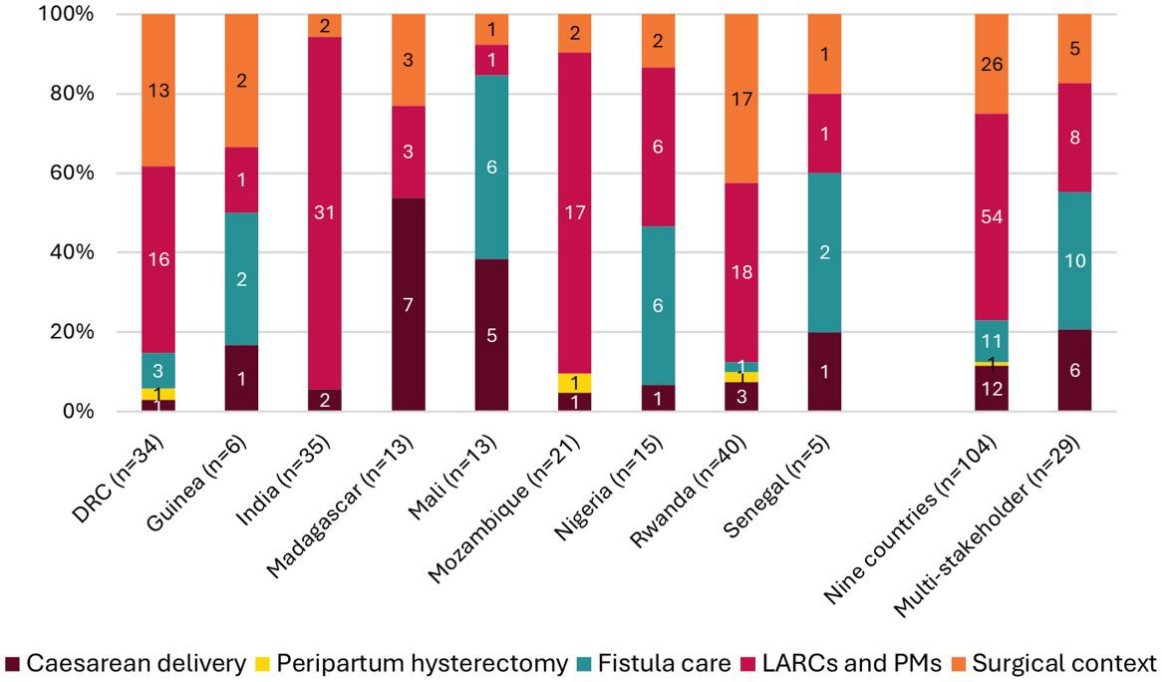
Number and relative distribution of indicators, by topic. DRC, Democratic Republic of the Congo; LARCs, long-acting reversible contraception; PMs, permanent methods of contraception.

Across all nine countries, we identified a total of 104 unique eligible indicators. The number of included indicators differed across countries (Fig. 2). Countries where we sourced information on indicators rather than data elements had lower numbers of included indicators. Countries also differed in the relative distribution of indicators across the five indicator topics (Fig. 2). All country RHIS included at least one indicator related to caesarean delivery, LARCs and PMs, and the surgical context. Fistula indicators were included in all RHIS, except in India, Madagascar, and Mozambique. Peripartum hysterectomy indicators were only included in the RHIS of DRC, Mozambique, and Rwanda. In India and Mozambique, most of the eligible indicators (>80%) were related to LARCs and PMs. Compared to other countries, Mali and Madagascar had the highest percentage (38% and 54%) of indicators focused on caesarean delivery. Fistula indicators made up more than 40% of the identified indicators in Mali, Nigeria, and Senegal.

The identified indicators were generally clear and quantifiable; however, there were differences in their specificity. For example, some indicators were disaggregated by specific implant devices: ‘Post-partum Implanon NXT inserted’ and ‘Post-partum Jadelle inserted’ (Nigeria). Only some indicators defined time frames of interest, such as ‘Number of post abortion sterilizations (within 7 days of spontaneous or surgical abortion) conducted’ (India).

Similar to our findings for the multi-stakeholder indicators, we observed that indicators across countries were often similar in meaning but phrased differently. For example, we identified three different indicators on insertion of postpartum intrauterine devices (IUD; which we included as one indicator in the analysis): ‘Number of postpartum (within 48 hours of delivery) intrauterine contraceptive device (IUCD) insertions’ (India), ‘Postpartum family planning uptake for copper [or hormonal] IUD’ (Rwanda), and ‘DIU no Pós-Parto Imediato [IUD in the immediate postpartum period]’ (Mozambique).

### Availability of multi-stakeholder indicators in countries’ routine health information systems

Out of the 29 eligible multi-stakeholder indicators, 14 (48%) were included in the RHIS of one or more of the nine countries. For the caesarean delivery topic, only one of the six multi-stakeholder indicators (17%) could be identified in countries’ RHIS (percent of deliveries by caesarean section). For fistula care, LARCs and PMs, and the surgical context, several of the multi-stakeholder indicators were identified in countries’ RHIS (fistula: 50%—5 out of 10, LARCs and PM: 50%—4 out of 8, surgical context: 80%—4 out of 5; for numbers by country see Supplementary Table S2).

**Figure 3.**
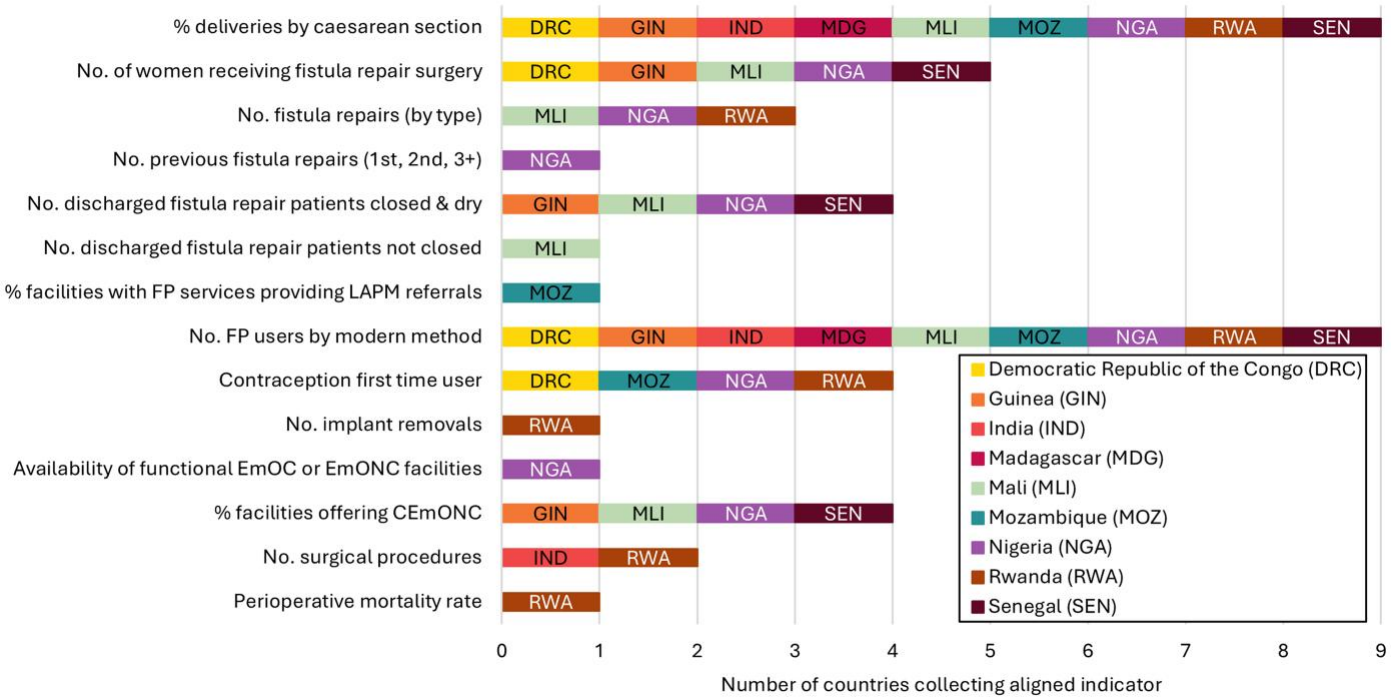
Overview of multi-stakeholder indicators which were identified within countries’ RHIS. CEmONC, comprehensive emergency obstetric and newborn care; EmOC, Emergency Obstetric Care; EmONC, Emergency Obstetric and Newborn Care; FP, family planning; LAPM, long acting and permanent methods of contraception; No., number.

Only two multi-stakeholder indicators were included in all nine country RHIS (percent of deliveries by caesarean section and family planning users by modern method of contraception). Four indicators were included in at least four of the nine countries (first time family planning users, women receiving fistula repair surgery, fistula patients discharged closed and dry, and healthcare facilities offering comprehensive emergency obstetric and neonatal care; Fig. 3).

Fifteen multi-stakeholder indicators were not found in any of the nine countries (Supplementary Table S1). These indicators were frequently related to facility readiness (e.g. availability of trained staff), procedure outcomes (e.g. post-operative complications), or specific subgroups (e.g. caesarean section rate among those with obstructed/prolonged labour). Highly specific multi-stakeholder indicators with a narrow scope were often absent from countries’ RHIS (e.g. post-caesarean severe systemic infection or sepsis). In some cases, broader country-level indicators related to similar concepts were present (e.g. post-operative infections following caesarean section; see Table 3). Although conceptually related, these indicators were not considered equivalent for the purposes of matching multi-stakeholder indicators to country RHIS indicators.

**Table 3.** Illustrative examples of country indicators not included on multi-stakeholder indicator lists.

Caesarean delivery	Peripartum hysterectomy	Fistula care	LARCs and PMs	Surgical context
Post-operative infections following caesarean sections (Rwanda, Mali) Caesarean delivery mortality (Mali, Madagascar) Deaths from post-caesarean infections (Rwanda) Caesarean sections with general, local, and regional anaesthesia (Madagascar)^a^ Caesarean sections performed at night, 8 p.m. to 8 a.m. (India)	Peripartum hysterectomies (Mozambique, Rwanda, DRC)	Post-rape urogenital fistula diagnosed and treated (DRC)^b^ Surgical status of women operated first, second and third time (Mali) Women discharged after fistula surgery (Nigeria) Women receiving a first repair (Nigeria) Women with vesicovaginal fistula managed medically (Mali)	Postpartum sterilizations (India, Rwanda) Postpartum intrauterine contraceptive device (IUCD) insertions (India, Nigeria, Rwanda, Mozambique) IUCD removals (India, Rwanda) Complications following IUCD insertion (India) Failures, complications, and deaths for female and male sterilization (India)^c^	Post-operative surgical site infections (India, Rwanda, Guinea, DRC) Surgical discharges during the month of which are authorized/cured, unauthorized, and referred (Rwanda, DRC) Anaesthesia, by type: gas, ketamine, general, spinal, regional, local (Rwanda, DRC) Obstetrical complications of anaesthesia: hospitalized and deaths (Rwanda) Days operating room is not functioning (DRC)

DRC, Democratic Republic of the Congo; IUCD, intrauterine contraceptive device; LARCs, long-acting reversible contraception; PMs, permanent methods of contraception; RHIS, routine health information systems.

^a^In the Madagascar summary form, anaesthesia is ‘disaggregated’ by general, local, and regional.

^b^In the summary form reviewed, this indicator appears to aggregate diagnosed and treated.

^c^The RHIS documentation reviewed shows failures, complications, and deaths disaggregated separately for both female and male sterilizations.

### Eligible country indicators not included on multi-stakeholder indicator lists

We identified 90 additional eligible indicators on country lists which were not included on the reviewed multi-stakeholder indicator lists. These covered all five indicator topics (Table 3). In contrast to the multi-stakeholder lists, we identified three countries (DRC, Mozambique, and Rwanda) with an indicator on peripartum hysterectomy in their RHIS. The two most common indicators (measured by four countries each) captured postpartum IUCD insertions and post-operative surgical site infections. The large number of additional indicators identified conveys that (i) countries’ RHIS included indicators on our surgical topics of interest despite their absence on the multi-stakeholder lists and (ii) indicators included on multi-stakeholder group lists differed from the indicators currently included within countries’ RHIS.

## Discussion

### Summary of main findings

To our knowledge, this landscape analysis is the first to collate, describe and compare RHIS indicators for surgical obstetrics and family planning. We identified 29 multi-stakeholder and 104 country indicators. There were marked cross-country differences with regards to the relative distribution of indicators across the five indicator topics, and multiple countries did not collect any indicators on fistula care or peripartum hysterectomy. There was little overlap between the identified multi-stakeholder and country indicators. Only 14 (48%) multi-stakeholder indicators were included in the RHIS of any of the nine countries, and only two (deliveries by caesarean section and family planning users by modern method of contraception) were included in all nine countries. We also noted gaps in the reviewed multi-stakeholder lists that were made evident by what was included in country RHIS (e.g. post-operative surgical site infections being measured in many countries). Country indicators were frequently counts of stock/equipment or of procedures, information which could be used to assess service readiness or coverage. However, measurement of post-surgical complications and other dimensions of quality (beyond surgical site infections) were more limited.

### Contextualizing study findings within the literature

The findings of our study concur with an earlier report on Health management information system (HMIS) data elements for maternal and newborn health, which focused on 24 low- and lower-middle income countries (Maternal and Child Survival Program 2018). The report found that while collecting the number of caesarean deliveries was common, data collection for other indicators varies considerably across countries. It also noted that certain labour and delivery indicators recommended for country and global monitoring were still not widely available in HMIS. Similarly, focusing on surgical obstetrics and family planning, our study revealed a lack of harmonization across countries and poor alignment with indicators put forth by multi-stakeholder groups. This could signal two interconnected issues: a lack of consensus guidance on what indicators to include in RHIS for surgical obstetrics and family planning, and limited use of indicator data at higher levels—a challenge well documented in the literature (Chanyalew *et al.* 2021, Hoxha *et al.* 2022, Qian *et al.* 2023, Yusuph *et al.* 2024). Lack of indicator standardization and harmonization are frequently cited barriers to RHIS data use (Wickremasinghe *et al.* 2016, Hoxha *et al.* 2022). By identifying gaps in indicator availability and alignment, our findings have the potential to inform technical strategies to improve RHIS data use for surgical obstetrics and family planning, such as initiatives focused on improving indicator design and simplifying tools for data capturing and reporting. This, in turn, can contribute to a self-sustaining cycle of improved data quality, increased data demand, and more effective use of information at all levels of the health system (Ikamari *et al.* 2007, Harrison and Nutley 2010).

We also observed variability in the number of eligible multi-stakeholder indicators across our five focus topics. For example, we identified ten fistula indicators, versus only six for caesarean deliveries and five for the surgical context. This variability may be the result of significant attention paid to standardizing fistula measurement as part of intense and high-profile investments into fistula elimination (Vogel *et al.* 2024). In contrast, measurement pertaining to caesarean delivery, which is the most common surgical procedure in women in low- and middle-income countries (LMICs) (Boatin *et al.* 2021), and other obstetric conditions have not (to our knowledge) received similar focused investment. The small number of surgical context indicators in global sources may have been related to efforts to streamline measurement to a minimum set of core indicators (Davies *et al.* 2021) and the relative recency of attention to global surgery within public health and development frameworks (Meara *et al.* 2015).

Our findings also reinforce the need to address some familiar challenges around indicator development and bottlenecks impeding the systematic collection and use of RHIS data (Benova *et al.* 2019). In looking at indicators put forth by multi-stakeholder groups or adopted by countries, we noted commonly reported issues such as duplication, vague indicator definitions, or difficult-to-quantify concepts. The WHO advises countries to adopt only a limited set of indicators (World Health Organization 2023); however, our analysis also illustrated that many national RHIS were overloaded with indicators. We initially sourced more than 700 potentially relevant country indicators, which sheds some light on the sheer volume of indicators that countries are attempting to measure through routine sources.

### Strengths and limitations

Our landscape analysis sourced indicators from published multi-stakeholder lists and national RHIS documentation from nine countries, allowing us to compare multi-stakeholder and country indicators and to describe cross-country patterns. There are previous examples of studies describing the inclusion and modification of multi-stakeholder indicators in country RHIS (Ngongo *et al.* 2015), but we extended beyond this by developing a novel and iterative approach which also allowed us to describe additional country RHIS indicators not currently on multi-stakeholder lists. Therefore, we were able to present a more comprehensive overview of available RHIS indicators for surgical obstetrics and family planning. Furthermore, the analysis’ strengths included methodologic flexibility that accommodated diverse types of data sources (e.g. HMIS monthly summary forms, metadata dictionaries, indicator lists). Lastly, we took steps to ensure that our screening process was as rigorous as possible (e.g. two reviewers, search algorithm).

The study's limitations include its narrow focus, the selective choice of source documents, and potential inconsistencies in data extraction and classification.

Our landscape analysis examined five indicator topics and was focused on nine countries which were connected to the MOMENTUM Safe Surgery project’s technical and geographic scope. We recognize that tracking high-quality surgery for obstetrics and family planning includes data on other procedures we chose not to focus on. Moreover, we also recognize the importance of reviewing what data exists for surgical procedures outside of surgical obstetrics and family planning (e.g. orthopaedics, paediatrics). Since we focused on surgical procedures, epidemiological indicators (e.g. number of diagnosed fistula cases) were excluded, even though they often appear on multi-stakeholder lists and in national RHIS. We chose to focus on specific surgical procedures to make this analysis manageable and topical to the project’s mandate. These findings may not necessarily generalize to countries that were not included in this study.

The multi-stakeholder indicators were sourced from 12 indicator lists developed by United Nations organizations or special initiatives related to the five indicator topics. We did not conduct a systematic review of all available multi-stakeholder consultations, which may have meant that relevant indicator lists from other sources were not included. The initial extraction of multi-stakeholder indicators was performed by a single reviewer, introducing the potential for unintentional omissions. In addition, applying the inclusion and exclusion criteria—as well as screening country RHIS indicators for alignment with multi-stakeholder indicators—was sometimes ambiguous and required a degree of subjective judgment, even with two reviewers involved and discrepancies resolved through consensus.

To source country RHIS indicators, we focused on indicator lists, metadata dictionaries and monthly summary forms. There may have been other data collection initiatives in country that included in-scope indicators, which were excluded in this analysis due to our focus on RHIS. To the best of our knowledge, our country-level sources were the most up-to-date as of December 2022, but we could not always confirm their completeness or account for changes after 2022. We did not verify if the identified indicators were actually included in the electronic RHIS platforms (e.g. DHIS2). Due to the variability of available source documents across countries, we could often only extract RHIS data elements, rather than calculated indicators. In some instances, comparing data elements to multi-stakeholder indicators may have led to overcounting (where we only checked whether the required numerator was present, not the denominator). In others, we may have undercounted the availability of multi-stakeholder indicators at the country level. For instance, we did not consider that some indicators could be calculated by summing multiple elements (e.g. surgical volume could be calculated if all individual procedures were included in the RHIS).

### Implications for practice

Our findings suggest that there is an opportunity to improve and streamline measurement of surgical obstetric and family planning data at country and global levels. Taking advantage of such an opportunity could help ensure that data are being measured and used appropriately and in service of improving health outcomes.

The multiplicity of global multi-stakeholder indicators suggests that indicator prioritization efforts may be occurring in isolation with limited coordination between actors. This observation was similarly reported in the maternal and newborn health context (Moller *et al.* 2018). Providing unambiguous, uniform, and actionable recommendations is also likely to aid the uptake of multi-stakeholder indicators as well as ensure that data is collected with a clear and defined purpose. The integrated nature of surgical obstetrics and family planning will require future indicator prioritization efforts to be led by a diverse group representing different disciplines. However, efforts to adopt additional indicators should go hand in hand with identifying and removing redundant or non-informative indicators to reduce the burden on data collection, and to improve data quality for use. Where procedures are not necessarily widespread, for example, inclusion of a global multi-stakeholder indicator in the RHIS may not necessarily be appropriate.

Moving towards RHIS that are fit for purpose also requires clear guidance on how data should be collected (e.g. at which level of the health systems, from what source documentation), who will collect and use the data, and how it should be interpreted. We found that identifying up-to-date data dictionaries and indicator definitions was challenging during the course of course of this study. This may indicate that this information is also not readily available to those who wish to use it, including district and facility managers. We found a substantial number of service readiness indicators (i.e. medicines, supplies, and equipment) which may be more appropriately captured in a logistics management information system or through discrete data collection activities such as health facility assessments or integrated supportive supervision activities. Moreover, while some of these indicators (e.g. stockouts of key commodities or days of operating theatres not functioning) were indicative of service readiness on their own, others (e.g. health professionals by cadre) would need to be co-interpreted with other indicators such as caseloads or volumes of procedures.

Lastly, there is an opportunity to strengthen the measurement of post-surgical outcomes and complications. Complications contribute greatly to adverse caesarean delivery outcomes, especially for emergency caesareans (Sobhy *et al.* 2019). Moreover, given the relative rarity of maternal mortality, expanding surgical morbidity measurement can support quality improvement efforts closer to real-time (e.g. during audit and feedback cycles), similar to what has been done with maternal near-miss case review (Lazzerini *et al.* 2018). As exemplified by the focus on severe maternal morbidity (Geller *et al.* 2018), many high-income countries have already invested in tracking health outcomes. In our study, we found that measurement of post-surgical outcomes and complications was still insufficient. Multi-stakeholder indicators on post-surgical outcomes and complications were rarely included in countries’ RHIS, potentially also due to countries prioritising indicators on other complications. For example, multiple country RHIS included an indicator on post-operative infections after caesarean, but none included the multi-stakeholder indicator on severe systemic infection/sepsis post-caesarean (World Health Organization 2016)—possibly because sepsis is a relatively rare maternal infection (Woodd *et al.* 2019). Consequently, our findings signal a clear need to expand and streamline measurement of post-surgical outcomes and complications as well as give clear examples of how they can be measured and interpreted.

### Persistent challenges and emerging lines of inquiry

Describing indicators intended for inclusion in national RHIS is a first step towards understanding the scope of measurement for surgical obstetrics and family planning. Our findings highlight gaps in these lists, including the frequent omission of indicators capturing peripartum hysterectomy or intra/post-operative complications. However, it is also important to assess whether listed indicators have been operationalized or remain aspirational by reviewing actual data availability within RHIS. This was beyond the scope of our study. As countries continue to expand indicator lists in these areas, attention must also be paid to data quality. Given that RHIS data are produced through a series of complex processes, multiple factors can compromise completeness and accuracy—ultimately limiting the ability of available indicators to generate valid and actionable insights (Bhattacharya *et al.* 2019, Moukénet *et al.* 2021, Shabani *et al.* 2025).

A common challenge is the inclusion of too many poorly defined, non-standardized, and often duplicative or overlapping data elements and indicators within RHIS (World Health Organization 2023). For example, studies in LMICs found maternal death causes were inconsistently reported across HMIS monthly summary forms and health facility registers (Maternal and Child Survival Program 2018, Kabue *et al.* 2023). Similarly, research conducted in hospitals across Bangladesh, Nepal, and Tanzania noted that labour registers contained essential data for key indicators, but health workers spent excessive time filling out complex, non-standardized forms (Shamba *et al.* 2021). In Bangladesh and Tanzania, health professionals were also burdened by duplicative RHIS tasks, often having to enter the same information multiple times across both paper-based and electronic systems (Ruysen *et al.* 2024). The availability of data capturing tools (e.g. registers, report forms, tally-sheets) varies across health system levels; those that are available are often underused, district-level data is often misaligned with registers, and complex indicators are frequently misreported (Rumisha *et al.* 2020). As electronic RHIS platforms (e.g. DHIS2) have expanded and measurement has become a growing focus, strengthening routine data quality and use becomes even more important—particularly as funding for household surveys such as Demographic and Health Surveys declines (Khaki *et al.* 2025).

These challenges are not unique to LMICs. In the United States, routine health monitoring is often fragmented. For example, pregnancy mortality surveillance is managed by the Centers for Disease Control and Prevention (CDC)’s Pregnancy Mortality Surveillance System (Centers for Disease Control and Prevention 2025), and surgical site infection monitoring is managed by both the American College of Surgeons’ National Surgical Quality Improvement Program or the National Healthcare Safety Network at the CDC (Ju *et al.* 2015). Outcome data typically come from administrative claims or electronic health records (EHRs). Although administrative claims data are structured, their focus on billing applications limits their utility for monitoring, as they capture certain events (e.g. sepsis, hysterectomy) well but miss more nuanced complications, such as severe anaesthesia reactions (Sigakis *et al.* 2016). Much of the relevant data exists in unstructured EHR clinical notes (National Healthcare Safety Network/Centers for Disease Control and Prevention 2025), which—similar to LMICs—often require manual abstraction to support decision-making. Natural language processing and large language models show promise in extracting complex outcomes, such as surgical site infections, postpartum haemorrhage subtypes (Bucher *et al.* 2020, Alsentzer *et al.* 2023), but such methods may not be feasible in many LMICs where EHR systems are limited. In LMICs, prioritizing a smaller set of indicators for aggregate reporting may be a more practical short-term strategy.

Consequently, more work needs to be done to inform the streamlining, standardization, and selection of essential indicators, and thereby to facilitate the measurement, analysis, and use of routine indicator data. Because we only looked at indicator lists and summary forms, the extent to which data for the indicators identified are actually being reported remains an open question. Variations in indicator documentation, e.g. by private versus public sector facilities, ought to be explored in future studies given the deficits in RHIS reporting observed of private sector facilities (Ohiri *et al.* 2023). It is not clear whether there are surgical obstetrics and family planning data captured in standardized facility registers that are not currently being reported in RHIS, for example via operating theatre logbooks. Identifying these data can inform future revisions in RHIS measurement or facility tools. It is also important to understand how routine data are being used in decision-making and quality improvement processes (e.g. during surgical audits, maternal death reviews, monthly facility meetings, etc.) and whether these data are being used in conjunction with other monitoring and quality improvement methods (e.g. Robson classification). Lastly, new indicators should be tested before being added to RHIS to ensure that they are clearly defined, measurable, needed, and actionable.

## Conclusion

Our landscape analysis identified a wide range of indicators for surgical obstetrics and family planning included within multi-stakeholder lists or the RHIS of nine countries. However, there are notable gaps as well as ample opportunities for streamlining and standardization. To generate actionable data on safe surgery within obstetrics and family planning, clear guidance is needed on what indicators to include in RHIS (i.e. prioritization), for whom they are intended (i.e. the audience), what they will be used for (i.e. the purpose), and how they should be collected (i.e. standardization).

## Supplementary Material

czaf052_Supplementary_Data

## Data Availability

The data underlying this article will be shared on reasonable request to the corresponding author.

## Reflexivity statement

The authors include 13 women and 5 men based across the Democratic Republic of the Congo, Guinea, India, Madagascar, Mali, Mozambique, Nigeria, Rwanda, Senegal, the United Kingdom, and the United States and with lived experience in other geographies. The author group is comprised of global public health practitioners and researchers with experience providing clinical care; implementing, monitoring, and evaluating surgical obstetrics, fistula care, and family planning programmes; designing routine health information systems; and assessing data quality and use.
