# Supplementary material for: Surgical indicators for obstetrics and family planning in routine health information systems: a landscape analysis
Source: Health Policy Plan. 2025 Aug 8;40(9):955–66. doi: 10.1093/heapol/czaf052 (PMC12516032; doi:10.1093/heapol/czaf052)
Supplement: czaf052_Supplementary_Data [file czaf052_supplementary_data.docx]

Supplementary Materials

Surgical indicators for obstetrics and family planning in routine health information systems: a landscape review

**Table of Contents**

[Indicator screening: inclusion and exclusion criteria 2](#_Toc181800664)

[Search terms 4](#_Toc181800665)

[Supplementary Figure 1 6](#_Toc181800666)

[Supplementary Table 1 7](#_Toc181800667)

[References for Supplementary Table 1 9](#_Toc181800668)

[Supplementary Table 2 10](#_Toc181800669)

# Indicator screening: inclusion and exclusion criteria

| **Inclusion** | **Exclusion** |
| --- | --- |
| Indicators directly related to the four focus procedures (1. Caesarean delivery, 2. Peripartum hysterectomy, 3. Fistula care, 4. LARCs and PMs). | Non-facility based interventions related to these procedures. For example:   - Social reintegration services after fistula surgery. |
| Indicators capturing general surgical context (even if not disaggregated by obstetrics).  Within this, indicators which captured obstetric-related conditions requiring surgery if identified were included:   - Uterine rupture - Ectopic pregnancy - Cervical tears | Maternal/perinatal health status indicators were excluded, unless disaggregated by obstetric surgery. For example:   - Postpartum complications - Stillbirth - Perineal tears   Indicators on the following procedures were excluded:   - Episiotomy - Manual removal of the placenta |
| Health outcomes of focus procedures (or surgery) were included. For example:   - Post-surgical complications - Morbidity and mortality related to surgery | We focused on clinical procedures and therefore generally excluded epidemiological dimensions (e.g. incidence, prevalence, or aetiology of health outcomes). For example, number of fistula cases was excluded but number of fistula repairs was included. |
| Indicators which captured readiness specific to the four focus procedures (+ surgery) were included. For example:   - Stocks of LARCs - Stocks of anaesthetics | Readiness indicators not specific to surgical procedures were excluded. For example:   - Stocks of antibiotics - Blood transfusion units/equipment |
| Indicators capturing general surgical readiness were included. For example:   - Surgical volume - Surgical length of stay - Surgical beds available - Surgical personnel | Generic readiness indicators were excluded. For example:   - Generic infection, prevention and control (e.g., water, gloves, handwashing kits) - Referral - Healthcare personnel - Infrastructure (e.g. sanitation, electricity) |
| Given our focus on RHIS indicators, we only included multi-stakeholder indicators which we considered measurable with RHIS data. For example:   - Indicators referring to facility policies - Indicators requiring a denominator which could be provided entirely via health services (e.g., % of women with a caesarean delivery receiving modern contraceptives at discharge) - Indicators measuring service provision (e.g., number of fistulas repaired) - Indicators measuring numbers of specific cadres of staff/staff with completed trainings | Multi-stakeholder indicators were excluded if we considered they were not measurable with RHIS data (e.g., were best measured via policy document reviews or surveys). Our exclusion criteria included:   - Indicators referring to national policies - Indicators with denominators requiring information about the entire population (e.g., % of all women using modern contraceptives) - Indicators requiring information on more than three dimensions because it was unlikely that such indicators could be measured without individual records (e.g., % of women in the health facility with a) post-partum haemorrhage b) due to a retained placenta c) for whom manual removal of the placenta was performed d) by a skilled birth attendant) - Indicators measuring quality because assessing quality is likely to require more than three dimensions (e.g., % of clients who receive high quality, comprehensive counselling for long-acting and permanent methods) - Indicators requiring modelling or linkage to other datasets (e.g., % of population with geographic access to facilities) |
| **General comments** | |
| Included indicators which required denominators (e.g., percentages) relied on counts of people using specific services (e.g., number of live births, women delivering, or women seeking family planning or fistula services). Where we only had information on data elements, we matched country data elements to global indicators based on the numerator alone (without checking whether the denominator was also available). | |

# Search terms

All search terms were used in conjunction with the Excel COUNTIF function.

| **English** | **French** | **Portuguese** |
| --- | --- | --- |
| *hysterectomy* | *hystérectomie* | *histerectomia* |
| *c-section*, *caesarean*, *caesarean*, *section* | *césarienne* | *cesariana*, *parto cirúrgico* |
| *Bellwether* | *Bellwether* | / |
| *EmOC*, *EmONC* | *SOUB*, *SOU-B*, *SOUC*, *SOU-C*, *SONU* | *EmOC*, *EmONC*, *cuidados obstétricos de emergência* |
| *emergency obstetric* | *urgence obstétrique* | *emergência obstétrica* |
| *Robson* | *Robson* | *Robson* |
| *obstructed lab* | *dystocique* | *trabalho de parto obstruído* |
| *VBAC*, *TOLAC* | *AVAC*, *EDTAC* | *TOLAC*, *VBAC* |
| *LAPM* | *LAPM* | / |
| *long acting*, *long-acting* | *longue-Durée*, *Longue Durée* | *ação prolongada* |
| *implant* | *implant* | *implante* |
| *permanent method*, * PM * | *méthode permanente*, * MP * | *permanent*, * PM *, *longa duração*, *irreversív* |
| *contracept* | *contracept* | *anticoncepcio*, *contracep* |
| *family planning*, * FP * | *planification familiale*, * PF * | *planejamento familiar*, * PF *, *planejamento reprodutivo*, *planeamento familiar* |
| *sterili* | *stérili | *esterilização* |
| *tubal ligation*, *tubal ring* | *ligature*, *trompes*, *tubair* | *tubár* |
| *LARC* | *CRLDA* | * LARC * |
| *IUD*, *IUCD*, *intrauterine* | *DIU*, *CDIU*, *intra-utérin* | *DIU*, *intrauterino* |
| *birth spacing* | *espacement des naissances*, *espacement des grossesses* | *espaçamento entre nascimentos* |
| *birth control* | *contrôle des naissances* | *controle de natalidade* |
| *injectable* | *injectable* | *injetável*, *injectàvel* |
| *Implanon*, *jadelle* | *implanon*, *jadelle* | *implanon*, *jadelle* |
| *vasectomy* | *vasectomie* | *vasectomia* |
| *fistula* | *fistule* | *fístula* |
| *first repair*, *second repair* | *première réparation*, *deuxième réparation* | *primeiro reparo*, *segundo reparo* |
| *operat* | *opérat* | *cirúrg*, *cirurg* |
| *obstetric* | *obstétrique* | *obstét* |
| *anesth*, *anaesth* | *anesth* | *anestés* |
| *blood* | *sang* | *sangue* |
| *surg* | *chirurg* | / |
| *perineal tear* | *déchirures périnéales*, *déchirure du périnée* | *laceração perineal* |
| *manual removal* | *l’expulsion manuelle*, *retrait manuel*, *extraction manuelle* | *remoção manual* |
| *destructive delivery* | *accouchement destructif* | / |
| *craniotomy* | *craniotomie*, *craniectomie* | *craniotomia* |
| *decapitation* | *décapitation* | *decapitação* |
| *retained placenta* | *rétention placentaire*, *rétention du placenta* | *placenta retida* |
| <>*test*, <>*smear*, <>*pressure* | <>*test*, <>*bilan*, <>*prise*, <>*frottis*, <>*pression* | <>*teste*, <>*esfregaço*, <>*pressão* |
| *soap*, *detergent* | *savon*, *détergent* | *sabão*, *detergente* |
| *transport* | *transport* | *transport* |
| *infection prevention*, *IPC* | *prévention des infections*, *PCI* | *prevenção de infecções*, *IPC*, *infecção hospitalar* |
| *infection control* | *contrôle des infections* | *controle de infecção* |
| *health worker* | *prestataire*, *agent de santé*, *travailleur de santé*, *agent sanitaire*, *travaille sanitaire*, *personnel* | *profissional de saúde* |
| *WASH* | *WASH*, *EAH*, *EHA* | * WASH * |
| *water* | *l’eau*, *d’eau* | *Água* |
| *bleach*, *chlorine* | *javel*, *chlore* | *alvejante*, *cloro* |
| *alcohol* | *alcool* | *Álcool* |
| *referral* | *référence* | *refer*, *transfer* |
| *antibiotic* | *antibiotique* | *antibiótic* |
| *glove* | *gant* | *luva* |
| *mortality* | *mortalité* | *mortalidade* |
| *death* | *décès* | *morte* |
| * MPDSR *, * MDR * , * MPDR * | *SDMPR*, *RDM*, *RPDM* | *vigilância do óbito materno e perinatal*, *auditoria do óbito materno e perinatal* |
| *confidential inquiry* | *enquête confidentielle* | *Inquérito confidencial* |

# Supplementary Figure 1

**Supplementary Figure 1: Indicator selection process**
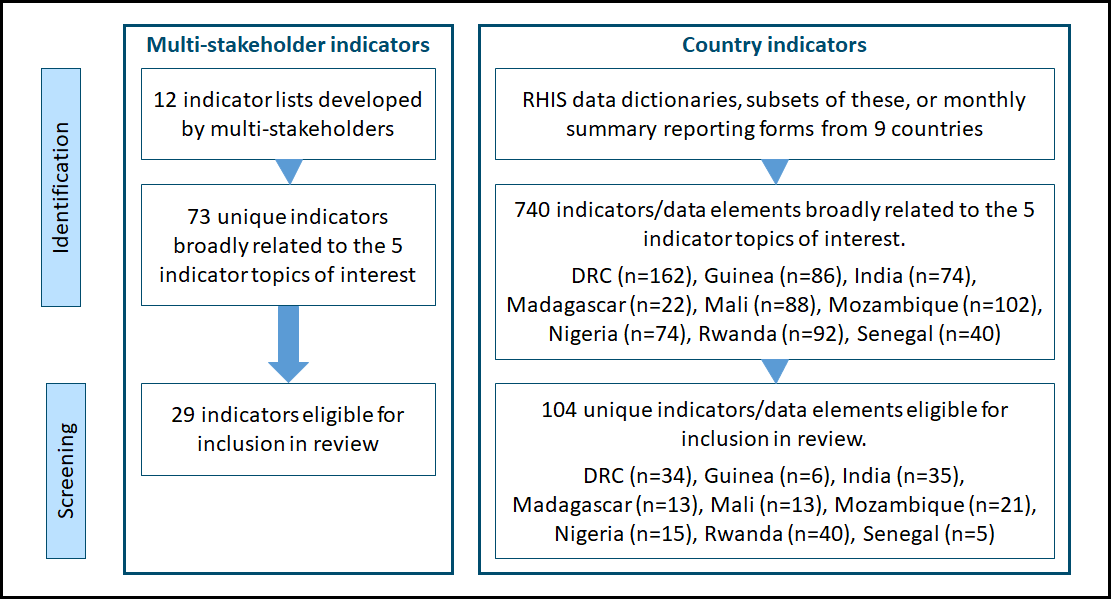


# Supplementary Table 1

**Supplementary Table 1: Eligible indicators put forth by multi-stakeholder groups and their availability across the RHIS of nine countries.**

| **Multi-stakeholder indicator name** | **DRC** | **Guinea** | **India** | **Madagascar** | **Mali** | **Mozambique** | **Nigeria** | **Rwanda** | **Senegal** |
| --- | --- | --- | --- | --- | --- | --- | --- | --- | --- |
| **CESAREAN DELIVERY (n = 6)** | | | | | | | | | |
| The health facility has an adequate number of staff skilled in performing caesarean section, 24 h a day.^1^ |  |  |  |  |  |  |  |  |  |
| Percentage of deliveries in health facilities by caesarean section.^2–6^ | ✓ | ✓ | ✓ | ✓ | ✓ | ✓ | ✓ | ✓ | ✓ |
| The proportion of all women in the health facility with prolonged and/or obstructed labour who gave birth by caesarean section.^1^ |  |  |  |  |  |  |  |  |  |
| The proportion of all women who underwent caesarean section in the health facility who received prophylactic antibiotics before caesarean section.^1^ |  |  |  |  |  |  |  |  |  |
| The proportion of all women who gave birth in the health facility by caesarean section who received a blood transfusion.^1^ |  |  |  |  |  |  |  |  |  |
| The proportion of all women who underwent caesarean section in the health facility who had severe systemic infection or sepsis after the caesarean section.^1^ |  |  |  |  |  |  |  |  |  |
| **FEMALE GENITAL FISTULA (n = 10)** | | | | | | | | | |
| Number of facilities with functioning obstetric fistula surgical treatment capacity.^4^ |  |  |  |  |  |  |  |  |  |
| Number of doctors trained in obstetric fistula repairs.^4^ |  |  |  |  |  |  |  |  |  |
| Number of women receiving fistula repair surgery.^8^ | ✓ | ✓ |  |  | ✓ |  | ✓ |  | ✓ |
| Number of fistula repairs by type (urinary-vaginal, rectovaginal fistula, combination).^8^ |  |  |  |  | ✓ |  | ✓ | ✓ |  |
| Number of previous fistula repairs (first attempt, second attempt, >2^nd^ attempt).^8^ |  |  |  |  |  |  | ✓ |  |  |
| Number of discharged fistula repair patients who are closed and dry.^8^ |  | ✓ |  |  | ✓ |  | ✓ |  | ✓ |
| Total number fistula repair surgery discharged/ number remaining in facility.^8^ |  |  |  |  |  |  |  |  |  |
| Number of fistula surgery complications by type (major surgical, anaesthesia related, postoperative complications related to perceived success of surgery, death).^8^ |  |  |  |  |  |  |  |  |  |
| Number of discharged fistula repair patients not closed or remaining with incontinence.^8^ |  |  |  |  | ✓ |  |  |  |  |
| Percent of women who have been treated for obstetric fistula who receive family planning or birth spacing counseling.^4^ |  |  |  |  |  |  |  |  |  |
| **LARCs AND PMs (n = 8)** | | | | | | | | | |
| Percent of facilities offering a permanent method of family planning.^4^ |  |  |  |  |  |  |  |  |  |
| Percent of service delivery points offering a mixture of short-acting modern contraceptive and long-acting reversible contraceptive methods.^4^ |  |  |  |  |  |  |  |  |  |
| Percent of facilities offering family planning services that provide referrals for LAPM.^4^ |  |  |  |  |  | ✓ |  |  |  |
| Percent of facilities with appropriate staff to support quality LAPM services.^4^ |  |  |  |  |  |  |  |  |  |
| Number of health providers trained in long acting and permanent services.^4^ |  |  |  |  |  |  |  |  |  |
| Number of family planning users by modern method of contraception at a defined point in time (contraceptive method mix)^7^ | ✓ | ✓ | ✓ | ✓ | ✓ | ✓ | ✓ | ✓ | ✓ |
| Contraception first time user: clients who accept for the first time in his/her life contraceptive method (by contraceptive method).^5,6^ | ✓ |  |  |  |  | ✓ | ✓ | ✓ |  |
| Number of implant removals: the number of contraceptive implants successfully removed.^4^ |  |  |  |  |  |  |  | ✓ |  |
| **SURGICAL CONTEXT (n = 5)** | | | | | | | | | |
| Number of functional emergency obstetric and newborn care facilities (EmONC) facilities.*^3,4,9,10^ |  |  |  |  |  |  | ✓ |  |  |
| Percentage of facilities offering comprehensive emergency obstetric and neonatal care (CEmONC).*^5^ |  | ✓ |  |  | ✓ |  | ✓ |  | ✓ |
| Percent of all births in EmOC facilities.^4^ |  |  |  |  |  |  |  |  |  |
| Number of surgical procedures undertaken in an operating theatre per year.^5,6,11^ |  |  | ✓ |  |  |  |  | ✓ |  |
| Perioperative mortality rate: all-cause death rate prior to discharge among patients that had one or more procedures in an operating theatre during the relevant admission.^5,11,12^ |  |  |  |  |  |  |  | ✓ |  |

* While some countries specified an EmONC indicator (which would include CEmONC and BEmONC), others measured its constituent CEmONC and BEmONC parts. If these were presented separately, we only selected CEmONC data elements/indicators and excluded BEmONC, even though these could have been combined to calculate a EmONC indicator.

## **References for Supplementary Table 1**

1. World Health Organization. Standards for Improving Quality of Maternal and Newborn Care in Health Facilities.; 2016. Accessed July 6, 2023. https://cdn.who.int/media/docs/default-source/mca-documents/qoc/quality-of-care/standards-for-improving-quality-of-maternal-and-newborn-care-in-health-facilities.pdf
2. World Health Organization. Mother and Newborn Information for Tracking Outcomes and Results: online indicator Toolkit (database). 2020. Accessed December 15, 2023. https://monitor.srhr.org/
3. Moran AC, Jolivet RR, Chou D, et al. A common monitoring framework for ending preventable maternal mortality, 2015-2030: Phase I of a multi-step process. BMC Pregnancy Childbirth. 2016;16(1). doi:10.1186/s12884-016-1035-4
4. MEASURE Evaluation. Family Planning and Reproductive Health Indicators Database. Accessed December 12, 2023. https://www.measureevaluation.org/resources/prh/rh_indicators
5. World Health Organization. Core Health Facility Indicators - Toolkit for Analysis and Use of Routine Health Facility Data Core Health Facility Indicators.; 2021. Accessed July 6, 2023. https://cdn.who.int/media/docs/default-source/world-health-data-platform/rhis-modules/facilityanalysisguidance-indicators-2021--01-21.pdf?sfvrsn=76b0be9b_5
6. World Health Organization, UNICEF. Analysis and Use of Health Facility Data: Guidance for RMNCAH Programme Managers.; 2019. Accessed July 6, 2023. https://cdn.who.int/media/docs/default-source/documents/ddi/facilityanalysisguidance-rmncah.pdf?sfvrsn=2055e453_2&download=true
7. FP2020. Core Indicators. Accessed July 6, 2023. http://www.track20.org/pages/data_analysis/core_indicators/overview.php
8. Ngongo C, Landry E, Levin K, Ndizeye S, Sutton I, Tripathi V. What to Measure and Why? Experience Developing Monitoring Indicators for an Emerging Maternal Health Issue: The Case of Obstetric Fistula. Vol 9.; 2015. www.jhidc.org
9. Every Woman Every Child. Global Strategy for Women’s, Children’s and Adolescents’ Health (2016–2030).; 2015.
10. Jolivet RR, Moran AC, O’Connor M, et al. Ending preventable maternal mortality: Phase II of a multi-step process to develop a monitoring framework, 2016-2030. BMC Pregnancy Childbirth. 2018;18(1). doi:10.1186/s12884-018-1763-8
11. Davies JI, Gelb AW, Gore-Booth J, et al. Global surgery, obstetric, and anaesthesia indicator definitions and reporting: An Utstein consensus report. PLoS Med. 2021;18(8). doi:10.1371/journal.pmed.1003749
12. World Health Organization. Global Reference List of 100 Core Health Indicators (plus Health-Related SDGs).; 2018. Accessed July 6, 2023. https://apps.who.int/iris/bitstream/handle/10665/259951/WHO-HIS-IER-GPM-2018.1-eng.pdf

# Supplementary Table 2

**Supplementary Table 2: Number of eligible multi-stakeholder indictors by topic and their availability across the RHIS of nine countries.**

|  | **Caesarean delivery** | **Peripartum hysterectomy** | **Female genital fistula** | **LARCs and PMs** | **Surgical context** | **Total** |
| --- | --- | --- | --- | --- | --- | --- |
| Multi-stakeholder indicators | 6 | 0 | 10 | 8 | 5 | 29 |
| Aligned indicators available in at least one country | 1 | *NA** | 5 | 4 | 4 | 14 |
| DRC | 1 | *NA** | 1 | 2 | 0 | 4 |
| Guinea | 1 | *NA** | 2 | 1 | 1 | 5 |
| India | 1 | *NA** | 0 | 1 | 1 | 3 |
| Madagascar | 1 | *NA** | 0 | 1 | 0 | 2 |
| Mali | 1 | *NA** | 4 | 1 | 1 | 7 |
| Mozambique | 1 | *NA** | 0 | 3 | 0 | 4 |
| Nigeria | 1 | *NA** | 4 | 2 | 2 | 9 |
| Rwanda | 1 | *NA** | 1 | 3 | 2 | 7 |
| Senegal | 1 | *NA** | 2 | 1 | 1 | 5 |

*The reviewed multi-stakeholder lists did not include any indicators on peripartum hysterectomy.
